# Syndecans as Cell Surface Receptors in Cancer Biology. A Focus on their Interaction with PDZ Domain Proteins

**DOI:** 10.3389/fphar.2016.00010

**Published:** 2016-02-02

**Authors:** Bill Cheng, Marine Montmasson, Laurent Terradot, Patricia Rousselle

**Affiliations:** ^1^Laboratoire de Biologie Tissulaire et Ingénierie Thérapeutique, UMR 5305, CNRS, Institut de Biologie et Chimie des Protéines, SFR BioSciences Gerland-Lyon Sud, Université Lyon 1Lyon, France; ^2^Bases Moléculaires et Structurales des Systèmes Infectieux UMR 5086, CNRS, Institut de Biologie et Chimie des Protéines, SFR BioSciences Gerland-Lyon Sud, Université Lyon 1Lyon, France

**Keywords:** syndecan, cancer, PDZ domain, phosphorylation, cytoskeleton, extracellular matrix

## Abstract

Syndecans are transmembrane receptors with ectodomains that are modified by glycosaminoglycan chains. The ectodomains can interact with a wide variety of molecules, including growth factors, cytokines, proteinases, adhesion receptors, and extracellular matrix (ECM) components. The four syndecans in mammals are expressed in a development-, cell-type-, and tissue-specific manner and can function either as co-receptors with other cell surface receptors or as independent adhesion receptors that mediate cell signaling. They help regulate cell proliferation and migration, angiogenesis, cell/cell and cell/ECM adhesion, and they may participate in several key tumorigenesis processes. In some cancers, syndecan expression regulates tumor cell proliferation, adhesion, motility, and other functions, and may be a prognostic marker for tumor progression and patient survival. The short cytoplasmic tail is likely to be involved in these events through recruitment of signaling partners. In particular, the conserved carboxyl-terminal EFYA tetrapeptide sequence that is present in all syndecans binds to some PDZ domain-containing proteins that may function as scaffold proteins that recruit signaling and cytoskeletal proteins to the plasma membrane. There is growing interest in understanding these interactions at both the structural and biological levels, and recent findings show their high degree of complexity. Parameters that influence the recruitment of PDZ domain proteins by syndecans, such as binding specificity and affinity, are the focus of active investigations and are important for understanding regulatory mechanisms. Recent studies show that binding may be affected by post-translational events that influence regulatory mechanisms, such as phosphorylation within the syndecan cytoplasmic tail.

## Introduction

**FIGURE 1:**
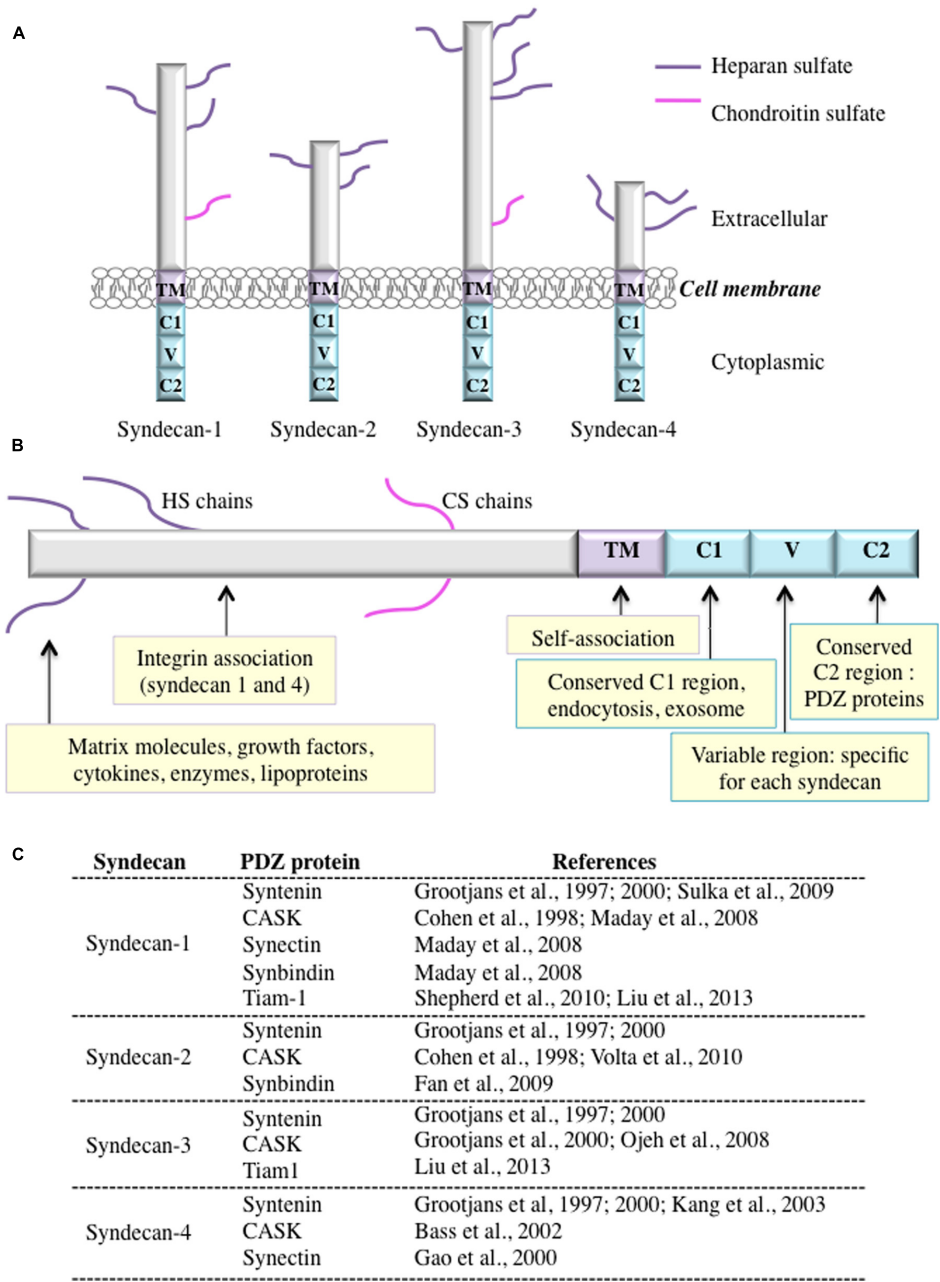
**(A)** The syndecan family of heparan sulfate proteoglycans. Syndecans consist of a transmembrane core protein with a short cytoplasmic tail and an ectodomain. All syndecan ectodomains are modified with either heparan sulfate only or heparan sulfate and chondroitin sulfate sugar chains. **(B)** A schematic showing the syndecan domains and examples of functions and interacting partners. **(C)** List of known PDZ-containing proteins that interact with syndecans.

Syndecans are transmembrane proteoglycans that are found on the surface of many types of mammalian cells. The four syndecans in mammals are encoded by four genes, but invertebrates have just one syndecan. Based on chromosomal localization and exon organization studies, all syndecans arise from a single ancestral gene. Syndecans are expressed in a development-, cell-type-, and tissue-specific manner and function either as independent or co-receptors that mediate cell signaling (Bishop et al., 2007; Multhaupt et al., 2009). In these type I transmembrane glycoproteins, the core protein ranges in size from 20 to 45 kDa. Syndecan core proteins include an extracellular domain (ectodomain) that carries either heparan sulfate only or heparan sulfate and chondroitin sulfate a single transmembrane (TM) domain; and a short cytoplasmic domain (**Figure 1A**). The ectodomain can interact with a wide variety of molecules, including growth factors, cytokines, proteinases, adhesion receptors, and ECM components. Syndecan-1 is mainly expressed in mesenchymal and epithelial cells. Syndecan-2 is highly expressed in endothelial and mesenchymal tissues and in liver, neural, and fibroblast cells. Syndecan-3, the longest of the four syndecans, is expressed in neural tissue and developing musculoskeletal system, but is undetectable in epithelial cells. Finally, syndecan-4, which has the shortest core protein, is widely expressed.

Syndecans are implicated in the control of cell–cell, cell–pathogen, and cell–matrix interactions via the recruitment of the actin cytoskeleton, as well as in cellular proliferation, differentiation, and migration. Syndecans can be found in cell protrusions and focal adhesions, where they colocalize with actin (Granés et al., 1999; Berndt et al., 2004). Importantly, they can act as co-receptors of other cell surface receptors like growth factor receptors and integrins (Morgan et al., 2007; Couchman, 2010; Rapraeger, 2013). In this context, syndecans can bind, immobilize, concentrate, and induce conformational changes in growth factors, adhesion molecules, and other signaling molecules via their heparan sulfate chains, thus facilitating their receptor interaction. They can also protect ligands from activation or sequester them away from membrane receptors (Zimmermann and David, 1999; Alexopoulou et al., 2007).

Syndecans undergo regulated physiological shedding of their extracellular domain, a process that may be increased in pathological conditions, thereby allowing them to act as soluble effectors and/or antagonists (Kim et al., 1994; Manon-Jensen et al., 2010). In addition, syntenins/syndecans, in conjunction with the syntenin-binding protein ALIX, are likely to be involved in or to enhance exosome production (Baietti et al., 2012; Friand et al., 2015).

## Syndecans and Cancer

Syndecans are involved in cancers, infectious diseases, obesity, wound healing, and angiogenesis. As documented in recent reviews, they are considered key regulators of tumor progression (Barbouri et al., 2014; Couchman et al., 2015; Theocharis et al., 2015). In some cancers, syndecan expression may regulate tumor cell function and serve as a prognostic marker for tumor progression and patient survival. Syndecan-1 expression is dysregulated in a number of cancers, including head and neck, ovarian, breast, and colorectal carcinomas (Teng et al., 2012). Syndecan-1 acts as a tumor suppressor in MDA-MB-231 breast cancer cells (Hassan et al., 2013). Treating these cells with syndecan-1 small interfering RNA not only enhances β1-integrin and focal adhesion kinase activity, leading to increased cellular adhesion and migration, but it also improves cellular resistance to irradiation. A study on pre-invasive breast cancer revealed an inverse correlation between the expression of syndecan-1 and the pro-metastatic microRNA miR-10b, suggesting a potential novel mode of post-transcriptional regulation of syndecan-1 (Hannafon et al., 2011). Studies revealing the negative regulation of syndecan-1 by miR-10b and its pro-invasive consequences in human breast cancer cells, reported syndecan-1 as a new regulatory target of miR-10b (Ibrahim et al., 2012). Other studies revealed that syndecan-1 decreases cell migration in lung epithelium via activation of Rap1, which slows focal adhesion disassembly (Altemeier et al., 2012). Syndecan-1 also plays a role in squamous cell carcinoma collagen-mediated motility and invasion by modulating RhoA and Rac activity, suggesting that decreased syndecan-1 expression during carcinoma progression may enhance tumor cell invasiveness (Ishikawa and Kramer, 2010).

The presence of syndecan-1 is associated with favorable outcomes in both lung cancer and mesothelioma (Kumar-Singh et al., 1998; Anttonen et al., 2001), and the loss of syndecan-1 is a feature of hepatocellular carcinoma with high metastatic potential (Matsumoto et al., 1997). Low syndecan-1 expression correlates with gastric carcinoma invasion and metastasis (Chu et al., 2008). In contrast, studies have reported that high expression levels of syndecan-1 in breast carcinoma are associated with high histological grade, high mitotic count, large tumor size, c-erbB-2 over-expression, and estrogen receptor-negative status. These studies show that high syndecan-1 expression correlates with the most invasive breast carcinomas (Stanley et al., 1999; Barbareschi et al., 2003; Leivonen et al., 2004; Lendorf et al., 2011). Studies using an *in vitro* breast cancer model also suggest that syndecan-1 participates directly in tumor cell spreading and adhesion (Beauvais and Rapraeger, 2003). In prostate cancer, high syndecan-1 expression is a feature of biologically aggressive progression (Zellweger et al., 2003). As stated in recent comprehensive reviews, stromal expression of syndecan-1 may have negative prognostic value, and elevated serum levels of the shed syndecan ectodomain might also be a prognostic indicator (Gharbaran, 2015; Szatmári et al., 2015). Studies have revealed a mechanism by which syndecan-1 and -4 ectodomains, may capture and induce autophosphorylation of the tyrosine kinase receptors HER2 and EGFR respectively, leading to integrin mediated carcinoma cell migration (Wang et al., 2014, 2015).

Nuclear localization of syndecan-1 has been reported, suggesting that it may function as a transcription factor and therefore impact gene regulation affecting cancer pathogenesis (Brockstedt et al., 2002). In addition, heparanase and syndecan-1 may cooperate to drive growth factor signaling and to regulate cell behavior, thus enhancing tumor growth and dissemination (Ramani et al., 2013; Palaiologou et al., 2014; Roucourt et al., 2015). One study found that syndecan-4 inhibited breast carcinoma cell invasion (Beauvais and Rapraeger, 2003), and its expression in human breast carcinoma was described as being associated with good prognosis (Lendorf et al., 2011). In contrast, another study found that syndecan-4 expression correlated significantly with high histological grade and negative estrogen receptor status (Baba et al., 2006) and was therefore a marker of poorer prognosis. Furthermore, a study of pancreatic cancer showed that syndecan-2 was involved in perineural invasion of pancreatic adenocarcinoma cells (De Oliveira et al., 2012). Silencing syndecan-2 expression in these cells significantly reduced motility and invasiveness. Syndecan-2 is upregulated in breast tumors (Lim et al., 2015) and in colon carcinomas (Park et al., 2002; Ryu et al., 2009; Choi et al., 2010). In highly metastatic colorectal cancer cells, syndecan-2 expression is enhanced by fibronectin secreted by stromal cells (Vicente et al., 2013). In colorectal carcinoma, low epithelial expression of syndecan-1 is associated with a higher histological grade, with more advanced clinical stage of the patients, and with potentially more unfavorable prognosis (Lundin et al., 2005; Hashimoto et al., 2008; Mitselou et al., 2012). Results from a recent meta-analysis of colorectal cancer studies demonstrated that loss of syndecan-1 expression in colorectal cancer correlates with histological grade and tumor stage, but not with lymph node or distant metastasis (Wei et al., 2015). The authors also reported that syndecan-1 expression does not have prognostic value in colorectal carcinoma patients. To date, syndecan-3 has not been implicated in cancer. Although the mechanisms are not yet fully understood, these examples highlight the important role of syndecans in tumor progression and suggest that they are relevant and promising therapeutic targets (Ramani et al., 2013; Barbouri et al., 2014; Theocharis et al., 2015). For instance, the anti-tumoral activity of zoledronic acid on breast cancer cells was reported to correlate with a differential modulation of syndecans (Dedes et al., 2012). Synstatin peptides based on HER2 and EGFR interaction motifs on syndecan-1 and -4 respectively can competitively displace receptor tyrosine kinase interaction and disrupt activation of cell motility (Wang et al., 2015). Similar peptides were designed to block IGF1R binding to syndecan-1/αvβ3 integrin complex and inhibit the integrin activity in endothelial and tumor cells (Rapraeger, 2013).

## The Transmembrane Domain-Induced Oligomerization Properties of Syndecans

Syndecans transmembrane domain is composed of 25 hydrophobic amino acid residues responsible for the molecular interaction that causes homo-oligomerization of syndecan core proteins (Asundi and Carey, 1995; Choi et al., 2005), a step essential for their signaling activation. The conserved GXXXG (where X is any amino acid) motif is involved in this process. Recent studies have revealed the potential of syndecan-2 and -4 to form hetero-oligomers, reducing each syndecan activity (Choi et al., 2015). This hetero-oligomerization capacity may offer insight into an underlying modulating mechanism (Kwon et al., 2015).

The cytoplasmic tail has two conserved regions, C1 and C2, that share common characteristics in all syndecans, plus a central variable region (V) that may regulate cell spreading and actin cytoskeleton assembly (**Figure 1B**; Carey et al., 1996; Chakravarti et al., 2005; Stepp et al., 2015). Each region can support signaling complexes formation (Carey, 1997; Bernfield et al., 1999; Yoneda and Couchman, 2003). The C1 domain is thought to participate in syndecan dimerization (Oh et al., 1997) and in the binding of various intracellular proteins, such as ezrin (Granés et al., 2000). In neuroblastoma, the C1 region of syndecan-3 interacts with a protein complex composed of Src family kinases and the actin-binding proteins cortactin and tubulin (Kinnunen et al., 1998). Likewise, the V region of syndecan-4 interacts with PKCα (protein kinase Cα) as well as with phosphatidylinositol 4,5-bisphosphate (PtdIns-4,5-P2) (Oh et al., 1997, 1998). The C2 carboxyl-terminal tetrapeptide sequence present in all syndecans consists of the highly conserved tetrapeptide sequence Glu-Phe-Tyr-Ala (EFYA) (Bass and Humphries, 2002; Multhaupt et al., 2009; Rousselle and Letourneur, 2009).

## The Interactions of Syndecans with Cytoskeleton PDZ Domain Proteins

The EFYA sequence binds to PDZ domain-containing proteins, such as syntenin-1 (Grootjans et al., 1997) and CASK (Cohen et al., 1998), which may function as membrane scaffold proteins that recruit signaling and cytoskeletal proteins to the plasma membrane. The EFYA motif thus belongs to the large family of PDZ-binding motifs (PDZ-BMs). Recent work suggests that PDZ interactions are involved in protein trafficking, possibly routing proteoglycans to the cell surface (Wawrzyniak et al., 2012).

There is growing interest in understanding the binding of syndecans to their PDZ domain-containing counterparts (**Figure 1C**). Not only are the interactions involved in cytoskeletal rearrangements in response to the signaling activities, but syndecan-PDZ domain-containing protein complexes may also participate in cell-ECM adhesion and migration. For example, synectin binding to syndecan-4 may modulate in vitro cell migration (Gao et al., 2000; Tkachenko et al., 2006). As well, cell adhesion to fibronectin is regulated by the interaction of syndecan-1 with the PDZ domain of the T-cell lymphoma invasion and metastasis gene 1 protein (Tiam1) (Shepherd et al., 2010). A study of hippocampal neurons revealed that syndecan-2 induces spine formation by recruiting intracellular vesicles toward postsynaptic sites through an interaction with synbindin (Ethell et al., 2000).

The name PDZ is an acronym derived from the first three proteins in which these domains were identified: PSD-95 (*postsynaptic density PSD-95/SAP90*), DLG (*Drosophila melanogaster tumor suppressor septate junction protein* Disks large-1), and ZO-1 (*epithelial tight junction protein* Zonula Occludens-1) (Kennedy, 1995; Zimmermann, 2006; Ye and Zhang, 2013). Over 250 non-redundant PDZ domains have been identified in the human proteome (Wang et al., 2010) and are found in proteins involved in diverse cellular functions, such as maintenance of cell polarity, signal transduction in neurons, and cell migration (Harris and Lim, 2001; Sheng and Sala, 2001; Jeleñ et al., 2003).

**FIGURE 2:**
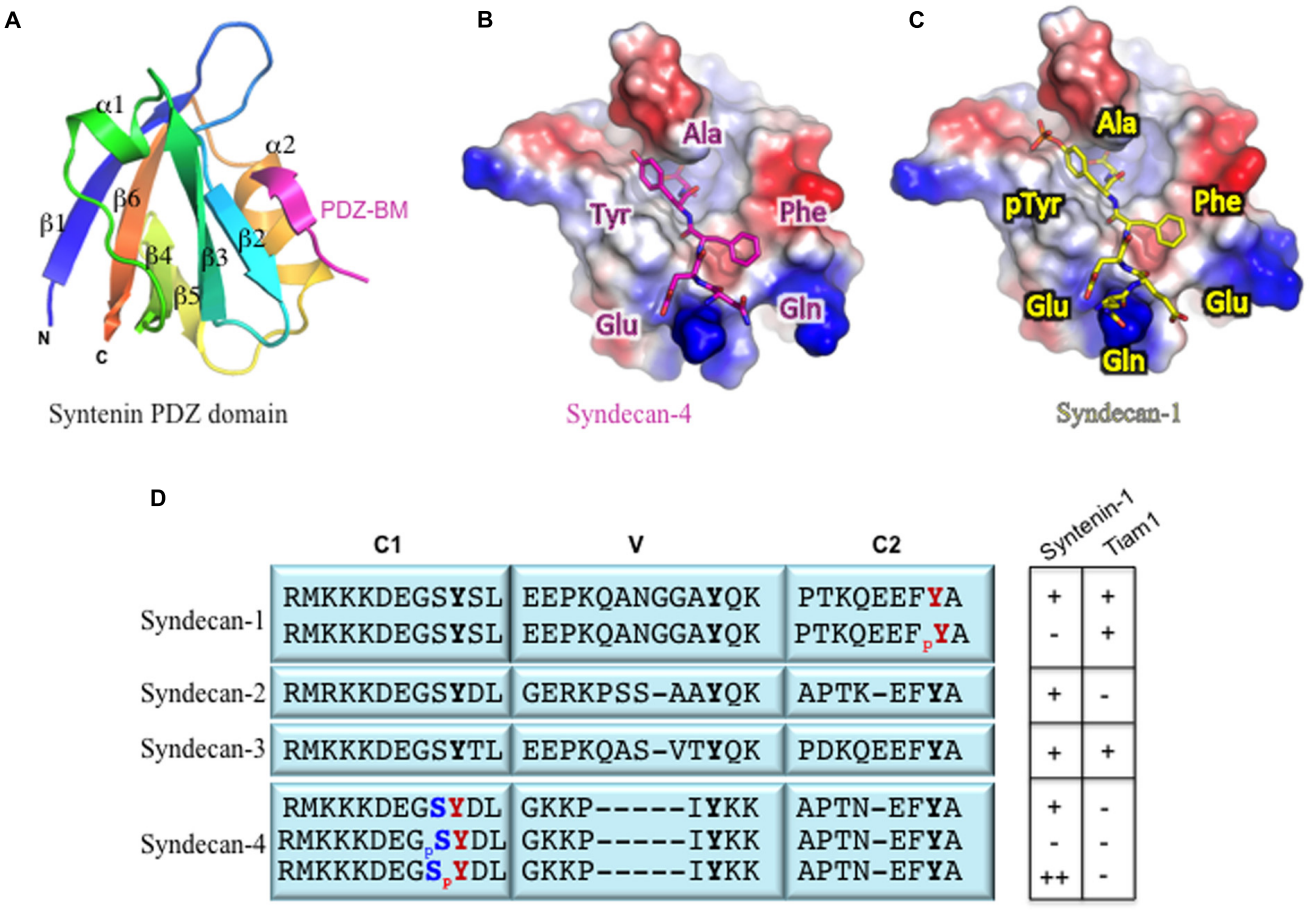
**(A)** Structural definition of syndecan binding to the PDZ domain. Ribbon diagram of the syntenin PDZ2 bound to the syndecan-4 peptide TNEFYA (pdb code 1 OBY, Kang et al., 2003), **(B)** Surface charge (blue positive and red negative) representation of the PDZ2 domain with syndecan-4 (magenta) peptide displayed as ball-and-stick. **(C)** Similar representation of PDZ2 with a model of syndecan-1 tyrosine phosphorylated peptide (yellow) showed as ball-and-stick. **(D)** Phosphorylation of Tyr and Ser residues within syndecan cytoplasmic tails and their effects on PDZ protein binding: (-) no interaction; (+) interaction; (++) enhanced interaction. The conserved (C1 and C2) and variable (V) domain organization is from Couchman et al. (2015).

The number of amino acid residues in a PDZ domain is relatively small (80–100 amino acids) (Hung and Sheng, 2002). Structural analysis of these domains indicates that a canonical PDZ domain consists of five or six β-strands and two or three α-helices (Luck et al., 2012) (**Figure 2A**). In addition, the domain itself folds into a compact globular shape; this maintains the proximity of the N- and C-termini to each other on opposite sides of the PDZ-BM interaction site (Sheng and Sala, 2001; Jeleñ et al., 2003; Lee and Zheng, 2010). The PDZ-BM fits in the groove between the α2-helix and the β2-strand structure such that the α2-helix is anti-parallel to the β2-strand (**Figure 2B**). This interaction site is also known as the carboxylate-binding site because of the highly conserved carboxylate-binding loop at the end of the groove that connects the β1- and β2-strand structures: R/K-X-X-X-G-Φ-G-Φ (where Φ is a hydrophobic residue) (Sheng and Sala, 2001; Hung and Sheng, 2002; Lee and Zheng, 2010).

There are no reports of PDZ domains interacting with syndecans through motifs other than the EFYA sequence. Since the EFYA motif is the only PDZ-BM in syndecans, it seems likely that all four syndecans have similar binding affinity for the same PDZ-containing proteins. For example, all syndecans have similar affinity for the PDZ1-PDZ2 tandem domain of syntenin-1 (Grootjans et al., 2000). However, CASK has a higher affinity for syndecan-2 and syndecan-4 than for syndecan-1 and syndecan-3. The molecular mechanism underlying this difference in affinities is unknown. On the other hand, the PDZ domain of Tiam1 binds to a peptide corresponding to the last eight residues of syndecan-1 and -3, but not to those of syndecan-2 and -4 (Liu et al., 2013).

PDZ domain-containing proteins play essential roles in most aspects of cellular homoeostasis and are implicated in diverse aspects of tumor development and metastasis (Subbaiah et al., 2011). A number of studies have established that MDA-9/syntenin has a pivotal role in cancer development and progression, and suggest that it could be a tumor marker (Philley et al., 2016). Recent data indicate that in addition to its involvement in the migration and growth of tumor cells, syntenin appears to be involved in controlling the plasma membrane localization of active β1-integrin (Kashyap et al., 2015). A recent study showed that co-upregulation of CASK and syndecan-2 in colorectal cancer is associated with an unfavorable prognosis (Wei et al., 2014), suggesting that CASK could be a prognostic factor for colorectal cancer metastasis. Synbindin was shown to contribute to the aggressiveness of gastric cancer by activating the ERK signaling pathway (Kong et al., 2013), while synectin was shown to participate in pancreatic cancer growth (Muders et al., 2006). As a guanine exchange factor for Rac1, tiam1 involvement in cancer biology may be linked to its pivotal function in cytoskeletal dynamics (Vigil et al., 2010). One study reported that syndecan-2 regulates colon carcinoma cell migration through Tiam1-dependent Rac activation (Choi et al., 2010).

## Regulation of PDZ Binding by Phosphorylation of the Syndecan Cytoplasmic Tail

The phosphorylation of Ser, Thr, or Tyr residues in the syndecan cytoplasmic tail appears to be a key mechanism that regulates its interactions with PDZ domains (**Figure 2B**). We reported that the formation of membrane protrusions in cells plated on immobilized laminin α3 chain LG45 domain required the dephosphorylation of tyrosine residues in the cytoplasmic tail of syndecan-1 (Sulka et al., 2009; Rousselle and Beck, 2013). Further experiments demonstrated that phosphorylation of the Tyr residue in its EFYA sequence abolished its interaction with syntenin-1 (**Figure 2C**, Sulka et al., 2009). In contrast, phosphorylation of this Tyr residue did not affect the binding of the PDZ domain of Tiam1 (**Figure 2D**, Shepherd et al., 2010; Liu et al., 2013). It is not known whether this holds true for phosphorylated syndecan-3 as well. Based on the examination of other syndecan-binding PDZ domains, the PDZ domains of CASK and synectin are predicted to interact with Tyr-phosphorylated syndecan-1 in a manner similar to that seen in the Tiam1 PDZ-phosphorylated syndecan-1 complex. This mechanism may support syndecan signaling specificity (Liu et al., 2013).

Other regulatory mechanisms involving the phosphorylation of a Ser residue in the carboxyl terminus of PDZ-binding proteins may either disrupts or enhances interactions with PDZ domains (**Figure 2D**, Cohen et al., 1996; Matsuda et al., 1999; Hegedüs et al., 2003). Studies of syndecan-4 revealed that phosphorylation of the Ser residue in the C1 region induces a conformational change in the C2 domain, even though the phosphorylation site is 20 residues away and impedes the PDZ binding ability of syntenin-1 (Horowitz and Simons, 1998; Koo et al., 2006). Furthermore, phosphorylation of the Tyr residue of the syndecan-4 C1 region was shown to enhance syntenin-1 binding and to function as a molecular switch to regulate specific integrin recycling and coordinate focal adhesion dynamics (Morgan et al., 2013).

These findings reinforce the importance of residues upstream of the EFYA motif in the regulation of PDZ domain interactions with syndecans. To date, there are no reports of the phosphorylation of Thr residues in terms of regulation of syndecan binding to PDZ domains. The phosphorylation of Tyr versus Ser residues depends upon which enzymes are involved. For example, Src family kinases and Elk kinases are widely reported to be the enzymes responsible for the phosphorylation of the Tyr residues (Asundi and Carey, 1997; Morgan et al., 2013). In contrast, PKCs are the only enzymes that have been reported to be involved in Ser residue phosphorylation (Prasthofer et al., 1995; Oh et al., 1997; Koo et al., 2006). Moreover, PKC can only recognize the Ser residue in syndecan-2 and syndecan-3, but not those in syndecan-1 and syndecan-4 (Prasthofer et al., 1995). Similarly, endogenously phosphorylated Tyr residues were only found on syndecan-1 and sydecan-4 in B82 fibroblasts, although this cell line also expresses syndecan-2 (Ott and Rapraeger, 1998).

Since phosphorylation is a key mechanism in modulating the interactions of syndecans with cytoplasmic proteins, the process is expected to be tightly regulated and some proportion of syndecans in a cell are expected to be in a phosphorylated state. Indeed, studies have found endogenously phosphorylated syndecans in cultured cells (Asundi and Carey, 1997; Ott and Rapraeger, 1998; Bass and Humphries, 2002; Morgan et al., 2013). These results illustrate the high level of complexity underlying the syndecans “turn on and off” signals.

## Conclusion

The study of both the structural and biological aspects of the mechanisms underlying PDZ protein binding to syndecans is an exciting field of research. Due to their high level of complexity, the physiological significance of these interactions is not yet fully clarified; however, ongoing and future work will undoubtedly shed light on these important molecular complexes and their roles in cytoplasmic signaling pathways.

## Author Contributions

PR and BC wrote the manuscript. MM prepared **Figure 1**. LT designed structural models presented in **Figure 2**.

## Conflict of Interest Statement

The authors declare that the research was conducted in the absence of any commercial or financial relationships that could be construed as a potential conflict of interest.

## Abbreviations

ECM: extracellular matrix
GAG: glycosaminoglycan
IGF1R: insulin-like growth factor-1 receptor
PDZ: postsynaptic density-95/disc large protein/zonula occludens-1
PDZ-BM: PDZ binding motif
PKC: protein kinase C
